# Systemic Carbonic Anhydrase Inhibitors in Common Ophthalmic Diseases: A Scoping Review from A Clinical Standpoint

**DOI:** 10.1007/s40135-025-00332-x

**Published:** 2025-07-08

**Authors:** Shahin Hallaj, Wesam Shamseldin Shalaby, Sapna Sinha, Jonathan S. Myers, Reza Razeghinejad

**Affiliations:** 1 Glaucoma Service, Wills Eye Hospital, Sidney Kimmel Medical College, Thomas Jefferson University, 840 Walnut St, Suite 1140, Philadelphia, PA 19107 USA; 2 Division of Ophthalmology Informatics and Data Sciences, Hamilton Glaucoma Center and Viterbi Family Department of Ophthalmology, Shiley Eye Institute, University of California, San Diego, La Jolla, CA USA; 3 Tanta Medical School, Tanta University, Tanta, Gharbia Egypt

**Keywords:** Acetazolamide, Carbonic Anhydrase Inhibitors, Dichlorphenamide, Ethoxzolamide, Methazolamide

## Abstract

**Purpose of Review:**

This paper provides a detailed overview of systemic carbonic anhydrase inhibitors (CAIs) and their application in managing ophthalmic conditions, including glaucoma, cystoid macular edema, and idiopathic intracranial hypertension. Despite their recognized clinical benefits, the potential for severe systemic adverse effects often discourages clinicians from prescribing these agents. By examining the pharmacology, pharmacokinetics, safety profile, and clinical indications of systemic CAIs, this review highlights strategies to mitigate treatment challenges, particularly in patients with comorbidities and complex medical backgrounds.

**Recent Findings:**

New insights into the diverse roles and distribution of carbonic anhydrase (CA) isozymes have expanded our understanding of both the mechanisms and clinical implications of systemic CAIs. In the eye, CA-II and CA-IV are key contributors to aqueous humor production and ocular blood flow modulation, with dose-dependent improvements in choroidal perfusion observed upon enzyme inhibition. Beyond mechanism of action, tissue-bound drug concentration is a key factor in achieving maximal intraocular pressure (IOP) reduction. Moreover, some CAIs exhibit activity independent of CA blockade, such as inhibiting aquaporin-4. Clinical data further indicate that despite theoretical risks of systemic toxicity—particularly in individuals with comorbidities—severe adverse events are relatively rare.

**Summary:**

In specific ocular diseases, the use of systemic CAIs is vital for maintaining vision. Although systemic CAIs carry risk of systemic adverse events, with higher risk, the incidence of severe adverse events is low and in some studies were comparable to that of topical therapy. It's important to be aware of potential side effects and ensure the correct usage of oral CAIs, especially in individuals with other systemic diseases. A tailored evaluation of risks and benefits carried out for each individual, particularly in case of prolonged usage, should decrease the risk of adverse events.

## Introduction

Carbonic anhydrase enzyme (CA) plays a crucial role in regulating various physiological processes, including acid–base equilibrium, ion transport, pancreatic juice production, memory, muscular and neural function, and aqueous humor production [1, 2]. Its primary function is catalyzing the conversion of carbon dioxide and water into bicarbonate and hydrogen ions, thereby maintaining fluid balance and pH levels. CA I-IV are the most abundant among the 16 isozymes of the CA enzyme in the eye [3]. Carbonic anhydrase inhibitors (CAIs) were introduced in the mid-1900 s [4], and later became available in oral, intramuscular, intravenous, and topical forms. While widely recognized for their efficacy in lowering the intraocular pressure (IOP), are also used in certain types of the macular edema and idiopathic intracranial hypertension (IIH) [1]. Despite their significant efficacy, the use of systemic CAIs can be associated with severe side effects [5], posing challenges for clinicians in the decision-making process. This review covers the pharmacology, pharmacokinetics, indications, and side effects of systemic CAIs.

## Materials and Methods

A comprehensive search of Medline and PubMed databases was performed on articles published between 1960 and 2023 using the following keywords and combinations: carbonic anhydrase inhibitors, acetazolamide, methazolamide, intraocular pressure, mechanism of action, carbonic anhydrase, glaucoma, retina, macular edema, retinitis pigmentosa, idiopathic intracranial hypertension, side effects, sickling disorder, sulfa allergy, trabecular meshwork, pharmacology, pharmacokinetics, metabolites, pregnancy, epilepsy, diabetes, anticoagulants, liver disease, and renal impairment. Reference lists of the included papers were used to further guide the literature review.

## Carbonic Anhydrase Isozymes

Different CA isozymes present in the eye are shown in Table 1. Inhibition of CAs in the eye results in elevated tissue carbon dioxide pressure, extracellular acidification, and an intensified Bohr effect, leading to localized vasodilation. This may contribute to their dose-dependent effect on ocular blood flow [6–8]. It is believed that most of the ocular effects of CAIs are mediated through CA-II and -IV inhibition [9, 10].

**Table 1:** Different isoenzymes of carbonic anhydrase in the eye

Site		Expressed Carbonic Anhydrase Isozymes
Cornea	Endothelium	I, II, IV
Ciliary Body	Pigmented epithelium	I, II, III, IV
	Capillaries	I, II, IV
Lens	Epithelium and fibers	I, II, III, IV
Retina	Müller cells	II
	Cones	I, II
	Pigment epithelium	IV
Choroid	Choriocapillaris	I, II, IV
Ophthalmic Artery	Orbit	IV

The cytoplasmic CA-II isozyme is found in the retinal pigment epithelium (RPE), lens, corneal endothelium, iris, and ciliary processes. In the ciliary body, CA-II plays a vital role in aqueous humor production by producing bicarbonate and facilitating the active transport of sodium and chloride ions, establishing an osmotic gradient, and promoting water movement into the posterior chamber. The inhibition of this enzyme is achieved when more than 99.9% of it is bound to CAIs [11, 12]. CA-II is highly concentrated in the kidneys, and systemic CAIs use is commonly associated with a moderate degree of metabolic acidosis [13], which may be one of the mechanisms involved in mediating the ocular hypotensive effects of CAI [14, 15].

CA-IV is a membrane-bound enzyme that plays a role in regulating acid–base balance by facilitating the removal of excess acid from the retina into the choriocapillaris [16]. CAIs are predominantly found in peripheral retina cones, with 91% testing positive, while rods show no CA activity. CA-negative cones, likely blue-sensitive, are absent near the foveal center and display distinct morphological traits compared to CA-positive cones [17]. CAIs enhance the pumping function of RPE cells by altering the activity of ion transport mechanisms. Additionally, CAIs can improve choroidal blood flow and cellular adhesion. These effects are partially mediated through the modulation of CA-IV activity and other enzymes such as gamma-glutamyl transpeptidase [18–20].

Multiple isozymes of CA exist in the choroidal plexus and other parts of the brain [21]. CA-II is highly expressed in oligodendrocytes, choroidal plexus, myelinated tracts, myelin sheaths, and astrocytes [21]. CA-III is present in the choroidal plexus but has a lower catalytic activity compared to CA-II [21, 22]. Highly expressed CA-II contributes to cerebrospinal fluid (CSF) production by choroid plexus, affecting bicarbonate and sodium exchange. CA-XII is another isozyme involved in CSF production, which is expressed on the basolateral membrane of the choroid plexus epithelial cells in the brain [23].

## Systemic Carbonic Anhydrase Inhibitors

### Acetazolamide

#### Pharmacology

Chemical characteristics of acetazolamide (AZA) are displayed in Table 2. The reports on the ocular hypotensive effect of AZA through the reduction of aqueous humor formation date back seven decades [24, 25]. Besides its hypotensive effect through enzymatic inhibition, AZA can further decrease aqueous humor production by inducing systemic and intraocular acidosis [14, 15]. Oral AZA reduces IOP by 30% to 40% [26]. AZA is ineffective in lowering the IOP in individuals with CA-II deficiency [27]. AZA increases blood flow and velocity within the middle cerebral artery. It also reversibly inhibits aquaporin 4, demonstrating a carbonic anhydrase-independent effect on water exchange [28], which is particularly noteworthy given its expression in the brain and the retinal Müller cells [29]. However, reports on its effects on retrobulbar and retinal blood flow are mixed [38, 53, 54]. AZA also exhibits other clinical effects, including enhancing adhesion between the retina and RPE, facilitating fluid movement from retina to choroid, and subretinal fluid resorption [30, 31].

**Table 2:**
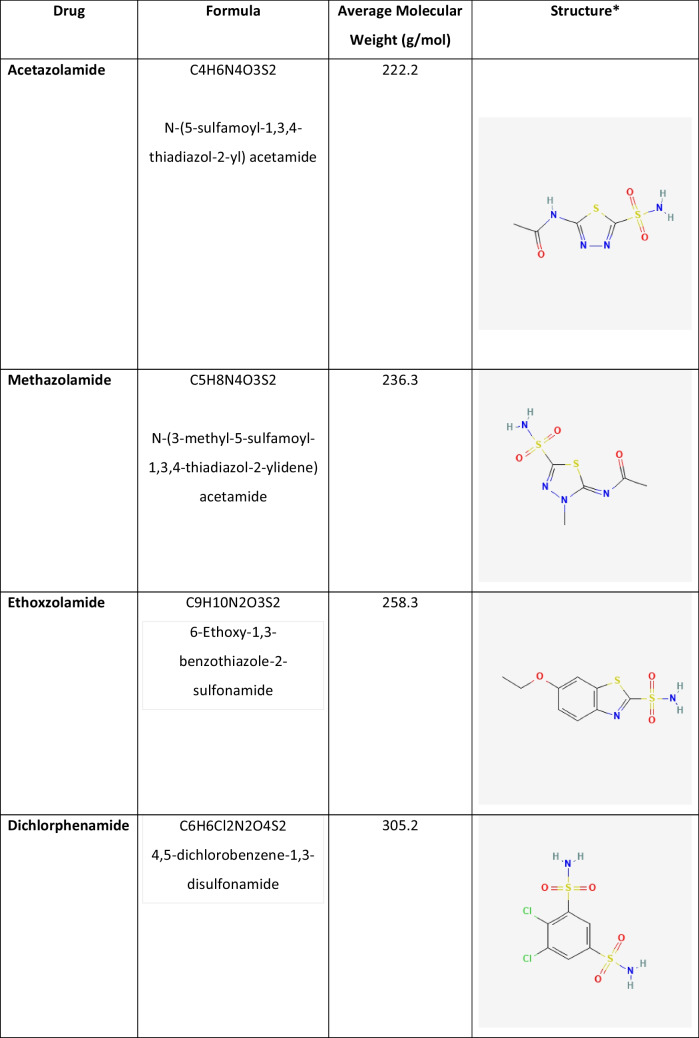
Chemical characteristics of systemic carbonic anhydrase inhibitors

*Two-dimensional chemical schemas were used from the https://pubchem.ncbi.nlm.nih.gov/ database

#### Pharmacokinetics

Pharmacokinetics of AZA is displayed in Table 3. It is well absorbed, with an oral bioavailability of 60–100%. Its maximum hypotensive effect is correlated with its tissue-bound concentration, not the plasma concentration, and is achieved when tissue-bound concentration approaches 99% of its maximum value [14]. The duration of action is inversely correlated with the drug's tissue dissociation rate. AZA has limited solubility in water, with 70–95% in a protein-bound form, mainly to albumin, limiting its distribution to the extracellular fluid. Despite its limited volume of distribution (0.2–0.3 L/kg), the drug reaches all organs, including the central nervous system [8, 32, 33]. AZA does not undergo metabolic alteration, kidneys excrete 70–100% of the administered dose. It is moderately dialyzable via hemodialysis (30% clearance with a 4-h hemodialysis). Peritoneal dialysis has limited clearance efficacy (less than 10% clearance over a 24-h dialysis).

**Table 3:** Pharmacokinetic information of systemic carbonic anhydrase inhibitors

Drug	Form	Onset of Action	Maximum Effect on IOP	Return to Baseline IOP	Peak plasma concentration	Traceable in Plasma	Serum Half-life
Acetazolamide	Tablets	1–1.5 h	2–4 h	4–12 h	1–4 h	8–12 h	2–6 h
	Slow-Release capsules	2–4 h	8 h	12–24 h	8–18 h	18–24 h	
	Intravenous Injection	5–10 m	15–30 m	4–6 h	15 m	4–5 h	
Methazolamide	Tablets	2–4 h	4–6 h	12–24 h	6–8 h	∼21 h	14 h
Ethoxzolamide	Tablets	2 m	5 h	12 h	N/A	N/A	6 h
Dichlorphenamide	Tablets	30 m	2–4 h	6–12 h	1.5–3 h	N/A	32–68 h

*IOP* Intraocular pressure. *h* hours. *N/A* not available

#### The Dose and Route of Administration

Different formulations of AZA are displayed in Table 4. The recommended dose of AZA for children is 10–20 mg/kg/day [34], and in adults, the maximum dose of 1000 mg/day. AZA is typically prescribed at dosages of 250 mg 4 times daily or 500 mg in sustained-release preparations twice daily [35, 36]. AZA sustained-release capsules taken twice a day are better tolerated and have a comparable IOP-lowering effect of tables taken four times a day. However, the cost of sustained-release capsules may be a limiting factor.2 The ocular hypotensive effect of AZA is dose dependent. In case of an emergency, intravenous or oral doses of 250–500 mg of AZA are administered. A 45% reduction in IOP can be achieved when the serum concentration of AZA reaches 15–20 μg/ml [36]. Some studies suggest that plasma levels greater than 10 μg/ml might not provide an additional hypotensive effect [37].

**Table 4:** Different formulations of systemic carbonic anhydrase inhibitors

Generic preparation	Other Names	Dosage	Protein Binding	Ka1 × 10^9^ (M)	pKa1	Solubility in water (mM)	Metabolism	Renal Excretion
Acetazolamide	Diamox tablets	125 mg 250 mg	95%	6	7.4	3	Minimal	100%
	Diamox sequels	250 mg 500 mg						
	Diamox parenteral	500 mg/vial						
Methazolamide	Neptazane	25 mg 50 mg	55%	8	7.2	5	75%	25%
	GlaucTabs							
	MZM							
Ethoxzolamide *(No more available in the market)*	Cardrase	125 mg 250 mg	95%	1	8.1	4	60%	40%
	Ethamide							
Dichlorphenamide	Daranide	50 mg	88%	18	8.3	N/A	Minimal	100%
	KEVEYIS							
	Ormalvi							

*Ka* Absorption constant. *pKa* degree of ionization. *N/A* not available

### Methazolamide

#### Pharmacology

Chemical characteristics of Methazolamide (MZM) are displayed in Table 2. MZM induces less metabolic acidosis, potentially explaining its milder ocular hypotensive effect [38]. At a 100 mg dose, MZM will bind to all CA-II isozymes and then to CA-I. The hypotensive effect of the drug occurs when the blood concentration reaches 2–4 μg/ml, achievable with a dosage of 50–100 mg twice daily [39–41].

#### Pharmacokinetics

Pharmacokinetics of MZM is displayed in Table 3. MZM exhibits slower intestinal absorption and blood clearance than AZA. Approximately 95% of the drug is reversibly bound to CA in red blood cells (RBCs). MZM has lower plasma protein binding (55%) and better distribution inside the eyes and central nervous system compared to AZA. The metabolic pathway of MZM is yet to be fully investigated, but studies suggest that hepatic enzymes such as cytochrome P450, glutathione, and β‐lyase are involved in its metabolism [40, 42–44]. Unlike AZA which undergoes up to 100% active renal excretion, only 25% of MZM is excreted unchanged in the urine, resulting in a longer half-life and requiring fewer doses [38, 45]. This makes MZM a suitable choice for patients with mild to moderate renal impairment and compromised glomerular filtration rate, as it is associated with fewer renal side effects and is preferable for individuals at high risk of kidney stones [39].

#### The Dose and Route of Administration

Different formulations of MZM are displayed in Table 4. MZM is available in 25 mg and 50 mg tablets and is used 2–3 times daily. To reduce the side effects, MZM may be started at 25 mg twice daily and then increased to 50 mg twice daily if needed [34, 38, 46]. The dosage can be escalated up to 100 mg three times daily. MZM at doses of 25, 50, and 100 mg three times daily may lead to reductions in IOP of 3.3, 4.3, and 5.6 mmHg, respectively [47]. To minimize the gastrointestinal side effects, it is recommended to take the medication after meals.

### Ethoxzolamide

#### Pharmacology

Chemical characteristics of ethoxzolamide are displayed in Table 2. Although ethoxzolamide is a potent systemic CAI in vitro, its high degree of protein binding and rapid uptake into adipose tissue reduce its effectiveness in vivo. These factors limit its bioavailability and therapeutic potential despite its strong in vitro enzyme-inhibitory properties [48].

#### Pharmacokinetics

Pharmacokinetics of ethoxzolamide is displayed in Table 3. Ethoxzolamide is well-absorbed from the gastrointestinal tract after oral administration. Its high protein binding and rapid uptake into adipose tissue, limits its availability in the systemic circulation which reduces its effectiveness. Ethoxzolamide is metabolized in the liver. The metabolic pathways and the exact metabolites have not been well-documented [48]. The primary route of excretion is through the kidneys, but a high proportion of the drug will remain in the adipose tissue for several months [48, 49].

#### The Dose and Route of Administration

Different formulations of ethoxzolamide are displayed in Table 4. However, they are no longer available on the market. Previously, ethoxzolamide was supplied in 125 mg tablet form and was prescribed in doses ranging from 62.5 to 250 mg every 4 to 8 h. Toxicology studies indicated that high doses could cause occasional gastric petechiae and hydronephrosis in rats, as well as potassium depletion in dogs [45, 48].

### Dichlorphenamide

#### Pharmacology

Chemical characteristics of dichlorphenamide are displayed in Table 2. As a CAI, it increases the urinary excretion of sodium, bicarbonate, and potassium, leading to metabolic acidosis. However, compared to AZA, it induces milder metabolic acidosis due to its inherent chloruretic activity [50]. Dichlorphenamide is more potent than AZA, being 30 times more effective in vitro and approximately 30% more effective in vivo for managing acute angle closure attacks [51]. One-third of the dosage of dichlorphenamide is required to achieve the same effect as AZA. But, dichlorphenamide may cause more symptoms and dose-dependent side effects compared to other CAIs [52, 53].

#### Pharmacokinetics

Pharmacokinetics of dichlorphenamide is displayed in Table 3, thought, there are limited studies on its pharmacokinetics. It is well absorbed orally and undergoes hepatic metabolism. Dichlorphenamide and its metabolites are primarily eliminated via the kidneys. Its effect tends to start earlier than AZA. Following oral administration of a 50 mg tablet, IOP drops within 30 min, reaches its lowest point within 2 to 4 h, then returns to baseline within 6 to 12 h [45, 51, 54]. Dichlorphenamide is a weak activator of hepatic enzymes such as cytochrome P450 [54–56].

#### The Dose and Route of Administration

Different formulations of dichlorphenamide are displayed in Table 4. Dichlorphenamide is available in 50 mg tablets. The initial recommended dose is 50 mg every 12 h, which can be increased to every 6 to 8 h, up to a maximum daily dosage of 200 mg. Although studies on dichlorphenamide use in glaucoma management date back to 1958 [53], it is not as widely prescribed as AZA [51].

## Indications In Ophthalmology

### Pediatric Glaucoma

Systemic CAIs are valuable medications in congenital glaucoma cases to lower the IOP prior to surgery [57]. They effectively reduce the IOP by 20–35% and may be taken with milk or food to improve tolerability. Compared to adults, CAIs may lead to a greater IOP reduction in pediatrics, which could be attributed to a decrease in CA activity with aging [58]. The pediatric dosage of AZA is 10–20 mg/kg/day, 2–4 times daily, while MZM dose is 2 mg/kg/day, twice daily [59]. One of the listed side effects of systemic AZA is growth retardation due to metabolic acidosis [59]. Given the children's limited ability to communicate, close monitoring of adverse effects is crucial. Oral sodium citrate (1 mEq/kg/day) has been used to treat the metabolic acidosis of systemic CAIs [60]. In a clinical study involving long-term (> 3 months) AZA use, no weight changes were observed in 22 children aged 2 months to 15 years [61]. However, any loss of appetite or weight loss should be monitored for early detection of potential problems [62].

### Adult Glaucoma

#### Acute Angle Closure

In acute angle-closure attacks, 250–500 mg of AZA is administered orally or intravenously and then every 4 to 6 h if needed. Intramuscular administration is allowed but is painful. In individuals with no prior history of renal dysfunction or electrolyte disorders, are used safely for few days/weeks [63, 64]. The use of an anti-emetic agent can enhance tolerance in cases of nausea and vomiting. After successful treatment of an acute angle-closure attack, the ciliary body responds to IOP spike by entering a transient hyposecretion phase, which can result in temporary hypotony. Consequently, systemic CAIs should be discontinued. However, patients may require continued use of systemic CAIs until receiving laser iridotomy or cataract extraction [63].

#### Open-Angle Glaucoma

Long-term use of systemic CAIs in open-angle glaucoma is not recommended due to their potential adverse effects. They are typically reserved for cases where topical agents fail to reduce IOP to the target level, or in patients who decline any form of surgical intervention despite having uncontrolled IOP with maximum tolerated medical therapy [65–69]. Dose-dependent reduction of IOP of 15–30% has been reported with systemic CAIs. However, a small percentage of individuals may not respond to CAIs [70]. Systemic CAIs are traditionally used as one of the first-line treatments in eyes with inflammatory glaucoma. In inflammatory glaucoma associated with corneal edema, systemic CAIs may be the best choice since absorption of topical medications is limited, and the topical CAIs may impact corneal endothelial cell function [71].

In long-term treatment with systemic CAI, the patients need to be monitored closely for potential adverse effects, and involving primary care physicians seems reasonable. Reduced survival of glaucoma patients on chronic oral AZA was reported in one study from Norway that included 1147 patients with primary open-angle glaucoma or pseudo exfoliative glaucoma [72]. AZA users were defined as patients who used the drug for at least 2 years and/or had left the hospital after their last visit with a prescription for daily dose of 250–500 mg AZA (*N* = 492). While the average patient longevity was 14.2 ± 8.3 years, in the multivariable COX proportional hazards model adjusting for the glaucoma type, age at diagnosis, gender, and year of diagnosis, AZA use was associated with reduced survival (HR = 1.152, *P* = 0.021) with an unclear reason [72].

Systemic CAIs may be used to prevent IOP spikes following intravitreal injections or cataract surgery. In open-angle glaucoma or glaucoma suspects, prophylactic AZA, 60–90 min before intravitreal injection, lead to a significantly lower IOP in the AZA group (mean: 15.7 mmHg, range: 8–21 mmHg), compared to the control group (mean: 20.6 mmHg, range: 11–46 mmHg) 30 min after injection [73]. A clinical trial investigated the effect of different IOP-lowering interventions on eyes receiving intravitreal bevacizumab by comparing AZA, topical brimonidine, anterior chamber paracentesis, and no treatment. Anterior chamber paracentesis prevented the IOP elevation following injection, whereas AZA and brimonidine caused a faster return to baseline IOP than the untreated group [74]. Eyes with primary open-angle glaucoma may experience marked IOP elevation 3–7 h after cataract surgery. An oral dose of 500 mg AZA, one hour before or 3 h after cataract surgery reduces the incidence of postoperative IOP elevations [75]. Following cataract surgery, short-term oral AZA offered better IOP control than topical dorzolamide 2% in preventing IOP spikes [76]. Similarly, patients who received AZA before vitrectomy or buckling surgeries had lower post-operative IOP (22.11 mmHg vs. 36.36 mmHg), and no adverse effects were noted [77].

**Fig. 1:**
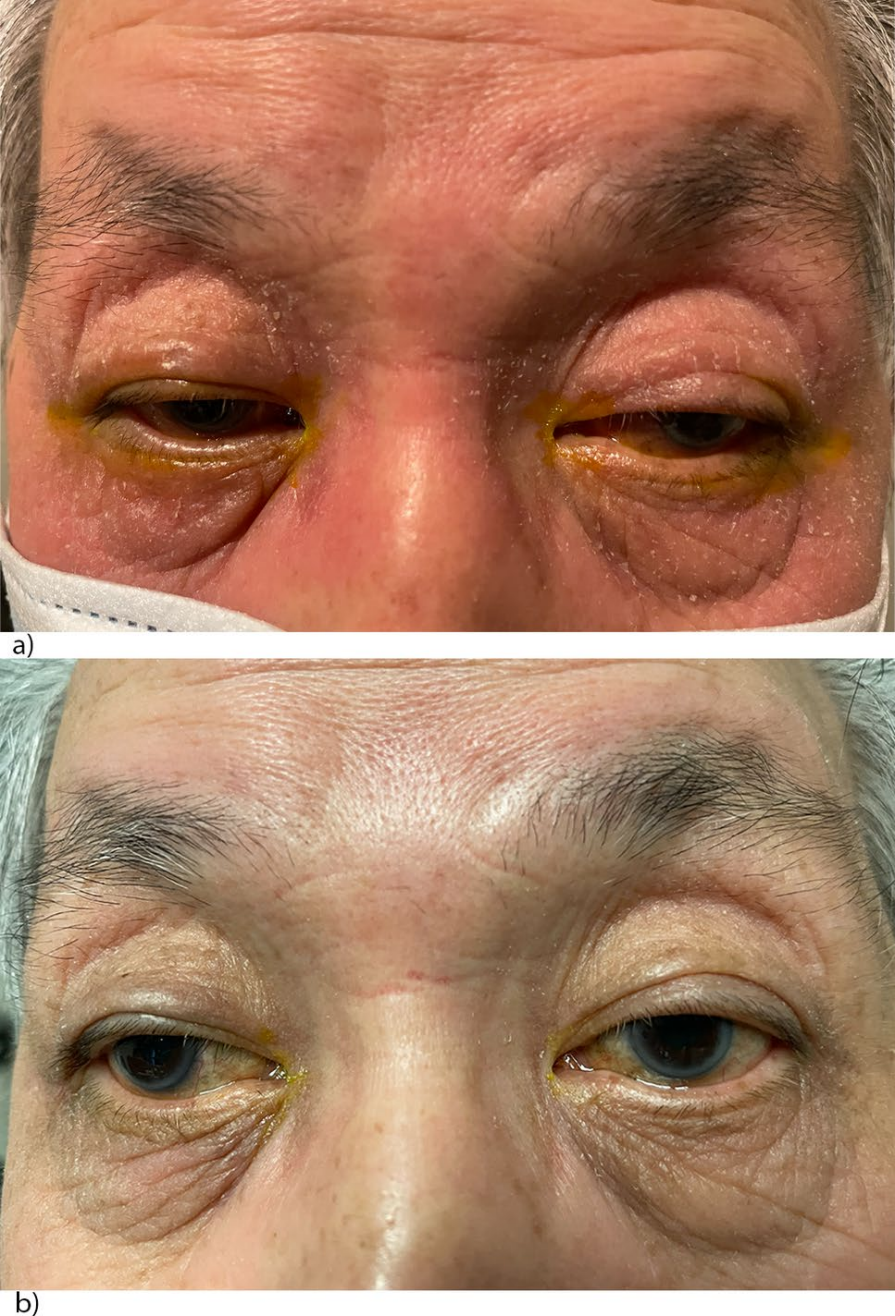
Figure 1: resolution of allergic contact dermatitis after discontinuation of the eyedrops and taking oral acetazolamide

In allergic contact dermatitis caused by glaucoma eyedrops, a potential management approach is discontinuing the eyedrops and initiating a short-term course of systemic CAIs. After approximately 3–4 weeks, the glaucoma eye drops can be reintroduced individually to identify which one was the cause of the allergic reaction. Alternatively, preservative-free glaucoma medications may be used, but the cost and their availability may be the limiting factor. This approach allows targeted identification of the causative agent and facilitates the implementation of appropriate measures to manage the patient’s condition effectively. Figure 1 illustrates the resolution of the allergic contact dermatitis induced by topical agents 3 weeks following the discontinuation of the eyedrops and taking oral AZA. Similarly, some glaucoma surgeons replace topical glaucoma medications with oral AZA and start topical steroids before trabeculectomy to decrease the conjunctival inflammation and reduce the incidence of bleb fibrosis and failure [78, 79]. It has been shown that long-term use of topical glaucoma medications can lead to histologic changes of the conjunctiva, with increased numbers and activity of lymphocytes, monocytes, fibroblasts, and conjunctival goblet cells. These changes ultimately contribute to bleb scarring [80, 81]. However, Lorenz et al. reported that preoperative, preservative-free, fixed combination dorzolamide-timolol was equally effective as preoperative oral AZA and topical dexamethasone in terms of IOP reduction and need for bleb needling or suture lysis. Notably, more patients reported adverse events in the AZA group than in the dorzolamide-timolol fixed combination group [82].

The effect of CAIs on intracranial pressure (ICP) has been well described [83]. According to Laplace’s law and stress formula, translaminar pressure depends on IOP, ICP, and lamina cribrosa. Some studies consider low ICP as a glaucoma risk factor [84, 85]. Loiselle et al. observed a significant reduction of ICP of 4 mmHg with an oral dose of 125 mg AZA in glaucoma patients but not in healthy subjects. However, this intervention did not affect the IOP in either group, but increased translaminar pressure in glaucoma patients [86]. CAIs can affect both ICP and IOP, but how this relates to clinical management and outcomes has not yet been determined.

### Retinal Diseases

CAIs are used in the management of cystoid macular edema (CME) and serous retinal detachment in patients with a variety of underlying pathologies, including retinitis pigmentosa, uveitis, Sturge-Weber syndrome, morning glory syndrome, optic disc pit, and central serous chorioretinopathy. CAIs affect CME by stimulating outward active transport and passive permeability across the blood-retinal barrier. AZA blocks the active transport of HCO3^−^ and Cl^−^ across the RPE and also increases the resorption rate of subretinal fluid. Acidifying the subretinal space and improving fluid hydrodynamics through RPE cells is another aspect leading to resolution of CME.

A common starting dose for AZA is 500 mg daily for a duration of 4 weeks. The dosage should be adjusted based on the patient's age and response to treatment [87]. Some uveitic patients may require a maintenance dose of 125 to 500 mg daily [88]. In cross-over trials on uveitic CME, patients taking a 4-week course of AZA showed a significant decrease in CME (25%) compared to placebo; however, no significant change in the visual acuity was observed [89].

AZA at the dosage of 500 mg/day for two weeks improved visual acuity in a patient with retinitis pigmentosa and chronic macular edema. Additionally, half of the patients had decreased angiographic CME, primarily due to less leakage [90]. MZM improved the angiographic CME in 9 of 17 retinitis pigmentosa patients. Although angiographic CME improved, notable or even moderate visual acuity improvement was observed in only four subjects [91]. In Yeo et al. study, the eyes with CME extending to the outer nuclear layer or central fovea, milder initial photoreceptor damage, and early treatment were more likely to have visual improvement. No significant changes were observed in microvascular parameters with treatment in those with visual improvement [92]. A systematic review and meta-analysis of 32 studies about CME management in retinitis pigmentosa found that topical and systemic CAIs and local steroids effectively reduce central macular thickness and improve visual acuity [93].

A study on the effect of oral CAIs showed that the time for subjective and objective resolution of serous retinal detachment in central serous choroidopathy was shorter with AZA compared to sham. However, the two groups had no difference in the final visual acuity or recurrence rate [94]. Rübsam et al. compared the efficacy of mineralocorticoid-receptor antagonists, AZA, and observation in central serous chorioretinopathy. Both mineralocorticoid-receptor antagonists and AZA were effective in decreasing the macular thickness and subretinal fluid with a corresponding improvement in visual acuity [95]. There is no definite evidence regarding the use of systemic CAIs in central serous retinopathies. Case reports showed the effectiveness of systemic CAIs in treating serous retinal detachment in Sturge-Weber syndrome, morning glory syndrome, and optic disc pit [96–99] Further research is needed to provide a comprehensive understanding of the optimal use of systemic CAIs in CME management [57].

### Idiopathic Intracranial Hypertension

Systemic CAIs are first-line treatments for the medical management of IIH, they lower the ICP by reducing CSF secretion [22]. A 99.5% reduction in CA activity in the choroid plexus is required to decrease the CSF production [100]. AZA is the only medication with level A evidence efficacy (well-conducted randomized controlled trials or meta-analyses of multiple randomized clinical trials with consistent results) to manage IIH. The resolution of papilledema starts after an average treatment period of 40 days, and the complete resolution may take up to 90 days [101].

Early studies suggested AZA-induced weight loss was the mechanism of the improved visual field, not the ICP reduction [102]. This was later disapproved by NORDIC Idiopathic Intracranial Hypertension Study Group trials. In a randomized, double-masked, placebo-controlled trial on 165 IIH patients with mild visual loss, participants were given a low-sodium weight-reducing diet and either AZA (up to 4 g daily) or a placebo for 6 months. The primary outcome was the perimetric mean deviation change. The perimetric mean deviation improvement was greater with AZA (1.43 dB) than placebo (0.71 dB). There was also improvement in papilledema grade and vision-related quality of life with AZA. The study reported that both the placebo (3.45 kg) and the AZA groups (7.50 kg) lost weight, obviously 4.05 kg more in the AZA group. Further analysis demonstrated that the benefit of AZA on perimetric mean deviation was not via its effect on weight, suggesting that the main mechanism of effect was through reduction of sodium ion transport across the choroid plexus with subsequent reduction of CSF production [103, 104]. In a randomized trial, 40 patients received AZA or topiramate and were monitored over 1 year. Both drugs showed statistically significant improvement of visual fields, with no significant difference between the two groups [105]. MZM could be used in patients with limited AZA tolerance or with renal and electrolyte disorders [57]. In addition to the improvement in papilledema, studies have reported the resolution of the sixth nerve palsy seen in IIH patients [106–108].

## Side Effects

Systemic CAIs, either for short- or long-term use, could be associated with several side effects attributed to the inhibition of CA enzyme, present in most tissues ranging from RBCs, renal tubules, gastric mucosa, pancreatic cells, eye, lung, testis, brain and bone osteoclasts (Table 5).

**Table 5:** Side effects of systemic carbonic anhydrase inhibitors

Ocular	Changes in color vision
	Induced myopia
	Secondary angle closure
	Choroidal effusion
Gastrointestinal	Dysgeusia
	Dry mouth
	Reflux and peptic ulcer disease
	Nausea and vomiting
	Diarrhea or constipation
	Abdominal cramps
	Liver Injury
Renal	Mild metabolic acidosis
	Alkaline diuresis ± Polyuria
	Nephrolithiasis
Hematologic	Blood dyscrasias
	Myelosuppression: thrombocytopenia, and aplastic anemia
Dermatologic	Steven-Johnson syndrome and toxic epidermal necrolysis
	Skin rash: Morbilliform or urticarial
Neurologic	Perioral and distal extremity paresthesia
	Malaise, fatigue, confusion, and drowsiness
	Anorexia and depression
	Headache, tinnitus, and vertigo
	Irritability, delirium, and insomnia
	Tremors
	Decreased libido
	Disturbed balance

### Ocular

Ocular adverse effects of CAIs include changes in color vision, transient myopia, secondary angle closure attack, and choroidal effusion [109, 110]. Disturbance of color vision is attributed to the inhibition of CA enzymes in the cones, resulting in decreased cone sensitivity to blue-green light [110, 111]. AZA-induced ciliary body edema causes forward displacement of lens-iris diaphragm, leading to myopia and secondary angle closure [112]. The onset of transient myopia can occur as early as 5 h to 5 days after taking the medication. Myopia resolves in a few days after cessation of the AZA [113].

### Gastrointestinal

CA-II enzyme is widely distributed in the epithelial layer of the gastrointestinal tract, bile duct, pancreatic duct, gall bladder, hepatocytes, and serous acinar cells of the parotid, submandibular, and gastric parietal glands, with the primary role of supplying bicarbonate ions to the secretions [114]. The vast range of gastrointestinal side effects include dysgeusia, dry mouth, reflux, nausea, vomiting, diarrhea, constipation, abdominal cramps, and peptic ulcer disease. AZA increases the odds of nausea, gastroesophageal reflux disease, and diarrhea by 2.6—4 folds [5, 115]. Oral administration of sodium acetate or sodium bicarbonate helps alleviate these symptoms in 50% of patients by partly correcting the CAI-induced metabolic acidosis [116, 117]. Epstein et al. reported that administering the drug with food relieves these gastrointestinal symptoms in about 50% of patients [116].

### Renal

CA-II enzyme is the predominant isoform of renal CA. It is expressed widely by multiple segments of the nephrons, including proximal convoluted, straight tubules, Henle’s loop, and intercalated cells of the cortical medullary collecting ducts [118]. Systemic CAIs induce mild metabolic acidosis and alkaline diuresis, presenting as polyuria in some patients. In normal individuals, the amount of diuresis is limited as the distal tubules reabsorb the filtered bicarbonate, fluid, and salts. In contrast, these symptoms are exacerbated in diabetics and those with chronic kidney disease, wherein the risk of metabolic acidosis is higher due to delayed drug clearance [119].

Prolonged administration of AZA may cause nephrolithiasis due to a decreased urinary citrate, resulting in the accumulation of calcium phosphate. Furthermore, an increase in urinary oxalate contributes to the formation of calcium oxalate stones [120]. Kass et al. reported 11 times increase in the incidence of nephrolithiasis in patients on AZA. The mean interval to stone formation was 14.4 months [121]. The formation of renal stones is not dose-dependent [122]. In patients with renal stones, it is essential to assess the renal function, weigh the risks, and exercise utmost caution.

### Hematologic

Systemic CAIs are occasionally associated with blood dyscrasias, myelosuppression, thrombocytopenia, and aplastic anemia [123–125]. This does not seem to be due to CA inhibition, but an idiosyncratic reaction, which is not dose-dependent, unpredictable, and delayed in onset [126, 127]. Keisu et al. reported 11 cases of AZA-induced aplastic anemia in patients aged 63–85 years over a period of 17 years with an estimated incidence of 1 in 18,000 and relative risk of 13.3 (95% confidence limits: 6.8- 25.3) [128]. Patients on long-term oral AZA should be counseled regarding symptoms and signs of bone marrow failure, such as easy bruising, frequent infections, and fatigue. Some recommend obtaining complete blood counts bimonthly during the first 6 months of therapy, followed by every 6 months thereafter [129].

### Dermatologic

#### Steven-Johnson Syndrome/Toxic Epidermal Necrolysis

SJS and TEN are rare skin diseases, presenting with extensive involvement of skin and mucus membranes triggered by certain drugs or infections. SJS/TEN forms a spectrum wherein SJS affects less than 10% of the skin, and TEN over 30% of skin (more severe and life-threatening). The estimated incidences of SJS, SJS/TEN, and TEN in the US are 9.2, 1.6, and 1.9 per million adults per year, respectively [130]. Drug-induced stimulation of cytotoxic and helper T-cells results in a type IV delayed hypersensitivity autoimmune reaction leading to epidermal necrosis. CAI-induced SJS/TEN results in more extensive cutaneous manifestations with frequent ocular complications compared to other drugs causing SJS/TEN [131].

AZA-induced SJS/TEN is associated with HLA-B59 in patients of Korean descent [132]. A similar association between HLA-B*5901 and MZM-induced SJS/TEN is seen in patients of Japanese and Korean descent [133–135]. An extensive exome-wide and HLA-based association study by Jiang et al. identified HLA-B*55:02 as a novel risk allele in Han Chinese. The specific haplotype is invaluable as a genetic predictor of MZM-induced SJS/TEN with a sensitivity of 89% and specificity of 96% [136]. With studies showing increasing evidence of association between HLA-B*5901 and HLA-B*5901-Cw*0102 haplotype and MZM-induced SJS/TEN in Korean and Hans Chinese population, genetic screening prior to starting MZM would be beneficial in these populations [137]. In a study on 8 Korean patients with MZM-induced SJS/TEN, the mean duration of drug exposure was 22.0 ± 16.9 days (range: 2–58 days). The patients received MZM at a dose of 50 mg/day for glaucoma and postoperative IOP spike management. The latent period to symptoms varied from 12 to 58 days, with a mean of 23.0 ± 15.7 days [138].

#### Skin Rash

Skin eruptions following systemic use of AZA and MZM have two distinct patterns, namely morbilliform and urticarial. The maculopapular rash has a bilateral symmetric distribution involving the trunk and extremities, which disappears within 2–3 weeks after discontinuation. The urticarial type presents with a pruritic rash and swelling of the trunk and extremities. The diffuse erythematous rash are round edematous papules or arcuate plaques [139]. Maculopapular exanthema and acute generalized exanthematous pustulosis are reported with AZA [140].

### Neurologic

CA enzyme present in neurovascular unit plays an important role in maintaining the pH, formation and distribution of CSF, and regulation of cerebral blood flow [141]. Systemic AZA causes perioral and distal extremity paresthesia which is the most common dose-dependent adverse effect. The reported incidence of paresthesia varies between 51–70%, with an increased incidence in patients on higher dosages. However, this is readily tolerated and is alleviated by oral administration of potassium chloride or reducing the dosage of AZA [5, 142].

Other systemic CAIs induced adverse neurological effects include malaise, fatigue, confusion, depression, drowsiness, dysgeusia, headache, vertigo, irritability, insomnia, tremor, tinnitus, decreased libido, and disturbed balance. AZA-induced metabolic acidosis may lead to lethargy and malaise, which improves with taking bicarbonate or acetate supplements. MZM-induced delirium presents as intermittent psychosis, lethargy, disorientation, and incontinence of bowel and bladder, which resolves within a week after discontinuation [143].

## Special Situations and Contraindications

### Pulmonary Diseases

AZA may be used as a respiratory stimulant in chronic obstructive pulmonary disease (COPD) with the goal of improving oxygenation, reducing carbon dioxide retention, and aiding liberation from mechanical ventilation and/or attempting to correct a metabolic alkalosis [144]. Accordingly, if a patient has chronic respiratory acidosis with metabolic compensation (mild alkalemia) at baseline, inducing additional metabolic acidosis may be acceptable and may even stimulate respiratory drive [145]. However, it is contraindicated in patients with acute COPD exacerbation as it may aggravate respiratory acidosis in the absence of metabolic compensation [145].

### Liver Diseases

Systemic CAIs induced liver injury is rare, few cases have been reported [146–148]. The mechanism of hepatic injury is presumed to be due to hypersensitivity reaction similar to the sulfonamides induced liver damage, and occurs days to weeks after administration. The severity of the hepatic injury ranges from mild, asymptomatic elevations of liver enzymes to a fulminant hepatitis [149]. Accordingly, systemic CAIs should not be used in patients with liver cirrhosis, while in mild liver disease, dose adjustment is necessary. Hepatic involvement and jaundice can also occur in cases of SJS but are generally overshadowed by the severe cutaneous reaction [149]. Corticosteroids’ role in altering the ultimate outcome of hepatic injury is unproven. Rechallenge should be avoided, and patients should be warned about any sulfa-containing medications in the future [149].

### Kidney Diseases

Systemic CAIs may cause electrolyte imbalances and are not recommended in patients with renal impairment. Advanced renal dysfunction is a contraindication to systemic CAIs use, as AZA is eliminated exclusively by the kidneys and its use induces bicarbonaturia and metabolic acidosis [150]. Taking sodium bicarbonate to offset the systemic acidosis of AZA may increase the risk of kidney stone formations [145]. Oral AZA is excreted unchanged thought the kidneys. Therefore, creatinine clearance (CrCl) directly influences its efficacy and the duration of action. The following dosages are recommended in renal dysfunction based on the CrCl: [145, 151–153] CrCl > 50 mL/min requires a full dose (250 mg four times a day or 500 mg extended-release capsule twice daily), CrCl 10 to 50 mL/min requires a half dose (250 mg twice daily), while AZA should not be used for CrCl < 10 mL/min.

The recommended dose for those on hemodialysis and peritoneal dialysis are 250 mg twice daily and 125 mg daily, respectively. If a patient is dialysis or has severe kidney disease, appropriate communication with the nephrologist is mandatory, with monitoring of central nervous system disturbances. The dose adjustment may reduce the IOP-lowering effect of AZA [145].

The metabolism of MZM (where only 25% of MZM is excreted unchanged in the urine) makes it a safer choice than AZA in patients with advanced renal disease (e.g., diabetic patients with neovascular glaucoma and expected diabetic nephropathy). Hypothetically, urolithiasis is also less likely with MZM than AZA due to less suppression of urine citrate and alkalinization, therefore, MZM is preferred to AZA in patients with a history of renal lithiasis [45].

### Sickling Disorders

Systemic CAIs not only promote hemoconcentration but also induce systemic acidosis which are important factors in erythrocyte sickling. Besides lowering the aqueous humor pH, AZA increases the concentration of ascorbic acid in aqueous humor which exacerbate the sickling. MZM may be more desirable as it creates less systemic acidosis than AZA. If a systemic CAI use is necessary in treating high IOP associated with hyphema in a sickle cell patient, MZM is the preferred agent [154].

### Pregnancy and Lactation

According to the US Food and Drug Administration, AZA and MZM are classified as a class C agent. This indicates observation of adverse effects on animal fetuses with lack of well-controlled studies in humans. As class C medications, AZA and MZM should be contraindicated in pregnancy, and prescribed only if the potential benefit justifies the risk to the fetus [155]. Congenital malformations, such as ectrodactyly, syndactyly, skeletal ossification disturbance, axial skeleton malformation, exencephaly, anophthalmia, microphthalmia, cleft lip/palate, and retarded incisor teeth development, have been reported in experimental animals [156–159]. However, animal studies have shown teratogenicity at doses over 10 times the recommended human dose.

In humans, there are no adequate and well-controlled studies in pregnant women on systemic CAIs [160, 161]. In an observational study of 101 women with IIH, AZA use before 13 weeks of gestation was not associated with any major complications in the offspring [161]. In the retrospective study of the Collaborative Perinatal Project (CCP), 50,282 mother–child pairs were evaluated, 12 had first-trimester exposure to the drug, but no anomalies observed. In a total of 1024 patients with any exposures (at any trimester), no major or minor fetal anomalies were observed [162]. On the other hand, case reports of congenital malformations of the offspring have been described which include conditions such as teratoma [163], ectrodactyly, syndactyly, oligodontia [164], Additionally transient metabolic side effects and electrolyte abnormalities in newborns, such as hypocalcemia, hypomagnesemia, and metabolic acidosis have been described [165, 166].

AZA is excreted into human milk in minimal quantities. The benefits should outweigh the risks when used during breastfeeding. The infant's estimated dose from a maternal dose of 1000 mg a day is less than 0.7% of the maternal weight-adjusted dose [167]. American, Canadian, and French professional guidelines consider CAIs acceptable in breastfeeding [168–170]. However, product manufacturers advise using with extreme caution in breastfeeding [167].

## Drug Interactions

The most common drug interactions of systemic CAIs are listed in Table 6.

**Table 6:** Drug interactions of systemic carbonic anhydrase inhibitors

Drug		Risks of Concomitant Use
Metformin		Lactic acidosis
		Poor glycemic control
Aspirin		Anorexia
		Tachypnea
		Coma
		Death
Anticonvulsants	Phenytoin Carbamazepine	Reduced metabolism and increased serum levels
	Topiramate	Metabolic acidosis
		Kidney stones

### Metformin

Metformin has emerged as the most widely prescribed antidiabetic medication in type II diabetes and also for weight reduction [171]. Lowering the hepatic glucose production by metformin may cause a shift from aerobic to anaerobic metabolism. This may increase lactate production, causing lactic acidosis [172]. Inhibition of CA enzyme is also associated with a decrease in hepatic glucose production. Significant reduction in circulating blood glucose and elevated lactate level in diabetic rats treated with AZA were observed [173]. Caution must be exercised in prescribing CAIs in patients taking metformin, as concomitant use of both medications may increase the risk of lactic acidosis and poor glucose control [145].

### Aspirin

AZA inhibits CYP3 A4 enzyme which enhances the adverse effect of aspirin. Additionally, salicylates appear to competitively inhibit the plasma protein binding and renal tubular secretion of AZA. Caution is advised when AZA and salicylate are used concurrently as severe adverse effects have been reported, including anorexia, tachypnea, coma, and even death, likely as a result of acute salicylate intoxication secondary to its reduced liver metabolism [145]. The adverse events can be more severe in renal insufficiency [174, 175]. Accordingly, patients with any degree of renal impairment should avoid taking AZA concomitantly with aspirin [175].

### Anticonvulsants

Systemic AZA is occasionally prescribed by neurologists as an adjunct therapy in epilepsy, as the AZA induced acidosis may reduce the spread of seizure leading to reduction of the number of attacks or even complete remission of seizures [176]. AZA may increase serum levels of anticonvulsants such as phenytoin or carbamazepine due to inhibition of CYP3 A4 metabolism [177], Consultation with the patient’s neurologist is recommended before prescribing AZA in epileptic patients. Additionally, caution should be exercised in prescribing AZA in patients taking topiramate. Because topiramate inhibits CA enzyme, and its concomitant use with CAIs increases the risk of metabolic acidosis and kidney stones [177].

## Tolerability, Adherence, and Monitoring

Once the need for systemic CAIs is confirmed, oral AZA can be started as long as there are no contraindications. Because there is no antidote to AZA, it is essential to emphasize to the patient to seek immediate care if there is an overdose. Symptoms include dizziness, confusion, loss of consciousness, and convulsions. The pharmacist should be consulted on medication reconciliation to ensure there is no drug-drug interactions or have the primary care physician involved before starting the medication and monitoring the patient.

Counseling the patient is important. Encouraging the patient to drink additional water and take a high-potassium diet (banana, avocado, raisins, apricots, beans and lentils, potatoes, winter squash, spinach, and broccoli) may be helpful even with short-term use. The risk of kidney stone formation with long-term use may be reduced by dietary intake of certain foods that induce a mild metabolic alkalosis, which may theoretically increase urine citrate and possibly lower the risk (citrate inhibits crystallization of calcium salt) [145].

Blood urea nitrogen to creatinine ratio, complete blood count, and serum electrolytes may be tested at baseline before starting systemic CAIs and monitored periodically; however, testing these values for all patients is a controversial topic, and there is limited evidence on the possible benefits. For at-risk patients such as individuals with recurrent renal stones, chronic kidney disease, and diabetic patients on metformin, electrolyte levels (potassium and sodium in particular) and blood gas analysis are recommended before starting the medication and 3–6 months thereafter. Monitoring liver enzymes may be considered as well, as hepatotoxicity is a reported rare adverse event [178].

The frequent systemic side effects associated with the use of systemic CAIs may adversely affect compliance. A study evaluated the tolerability and efficacy of dorzolamide in those who were intolerant to systemic CAI. Within 4 weeks of switching from systemic CAI to dorzolamide, the mean health assessment scores improved significantly in seven of the eight categories of the SF-36 (The Short Form 36 Health Survey Questionnaire) and did not change for the rest of the study, reflecting the negative impact of systemic CAIs on health-related quality of life [179]. A systematic review evaluated the quality of life in glaucoma and 3 other systemic diseases [180]. In glaucoma patients, the lowest scores in most categories originated from the previous study discussing drug-related adverse effects of systemic CAIs [179]. These findings suggest that long-term use of systemic CAIs is discouraged if there are alternatives. On the other hand, a population-based cohort study in Canada involving 128,942 matched patients over a 25-year period, showed that the absolute risk of a severe complicated adverse event of SJS, TEN, or aplastic anemia was 2.90 per 1000 patients for systemic CAIs, and 2.08 per 1000 patients for topical CAIs. This indicates that the risk of severe adverse reactions from systemic CAIs was low and comparable with topical medications [181].

## Expert Opinion

Systemic CAIs are a well-established treatment for various ocular conditions, particularly glaucoma. They are used in both pediatric and adult cases, often in combination with topical medications to temporarily decrease the elevated IOP prior to glaucoma surgery. They are also used in patients who do not tolerate or have contraindications to topical medications, refuse the surgical interventions, are at high risk of surgical complications, or have undergone multiple surgeries without achieving a satisfactory IOP control. In addition to IOP reduction, systemic CAIs are used in retinitis pigmentosa CME and to lower the ICP in IIH.

The systemic CAIs seems to be underutilized by ophthalmologist due to the concerns for severe adverse effects. Oral CAI had an absolute risk of 2.90 per 1000 patients of developing a life-threatening side effect compared to 2.08 per 1000 patients with topical CAIs. The observation of a directly proportional association in the risk of these events over time could reflects patients’ underlying baseline risk of sustaining these events [181]. Considering that oral CAIs are associated with a low incidence of serious adverse effects, their proper use and comprehensive review of systemic diseases and other medications the patient is taking should decrease the occurrence of severe adverse effects. Furthermore, the incidence of severe adverse events is low and in some studies were comparable to that of topical therapy. lack of cost‐effectiveness, limited test availability, and the rarity make widespread or “routine” testing for CA‐II deficiency impractical. Instead, clinicians may reserve CA‐II assays for those with strong clinical suspicion based on characteristic symptoms and family history.

A thorough review of the patient’s medication regimen, particularly anticonvulsants, salicylates, and metformin, is necessary to prevent potential drug-drug integrations. Additionally, initial laboratory tests are suggested to exclude the presence of co-existing conditions. It is beneficial to engage the primary care physician in monitoring and managing patients who have associated systemic comorbidities (such as lung, liver, or kidney diseases) or those who need long-term CAIs treatment to enhance comprehensive care coordination. Initiating treatment at a low dose with a gradual increase, if needed, can enhance patient adherence, and reduce the risk of side effects expected with maximum dosage.

Indeed, in situations where the potential benefits surpass the risks, such as in instances of vision-threatening elevated IOP or ICP, or CME not responding to other treatments, the patient should not be deprived of the sight saving effects of systemic CAIs. This approach aligns with a comprehensive and patient-centered care model, where individual patient factors and circumstances are paramount in decision-making. It’s important to monitor for potential side effects and adjust the treatment plan as necessary. Switching from AZA to MZM seems a reasonable approach for patients with kidney disease, instead of discontinuing systemic CAIs. In situations where patients have minor side effects, applying suitable management and adjusting the dosage might be enough, rather than discontinuing the treatment. It's crucial to perform a tailored evaluation of the advantages and disadvantages for each patient prior to and during the use of systemic CAIs treatment. The continuous monitoring ensures the therapy remains appropriate for the individual's specific situation.

## Key References

- Schmickl CN, Owens RL, Orr JE, Edwards BA, Malhotra A. Side effects of acetazolamide: a systematic review and meta-analysis assessing overall risk and dose dependence. BMJ Open Respir Res. 2020;7(1).
  - Findings from this study was crucial in understanding the side effects of carbonic anhydrase inhibitors.
- Wall, M.; McDermott, M. P.; Kieburtz, K. D., et al. Effect of Acetazolamide on Visual Function in Patients With Idiopathic Intracranial Hypertension and Mild Visual Loss: The Idiopathic Intracranial Hypertension Treatment Trial. JAMA. 2014;311(16):1641-1651.
  - Findings from this study suggest that addition of Acetazolamide to low sodium diet may provide modest improvement in the visual field in patients with idiopathic intracranial hypertension.
- Popovic, M. M.; Schlenker, M. B.; Thiruchelvam, D.; Redelmeier, D. A. Serious Adverse Events of Oral and Topical Carbonic Anhydrase Inhibitors. JAMA Ophthalmology. 2022;140(3):235-242.
  - This study suggests that adverse effects following systemic or topical carbonic anhydrase inhibitors use is rare and similar across different agents.

## Data Availability

No datasets were generated or analysed during the current study.
